# Liver Transplantation Without Systemic Antifungal Prophylaxis—An Exceptional Perspective from a Single Center Experience

**DOI:** 10.3390/jcm14134663

**Published:** 2025-07-01

**Authors:** Kenan Moral, Gökhan Kabaçam, Muzaffer Atlı, Mehmet Cindoruk, Yaşar Bayındır, Yeşim Sardan, Sedat Karademir

**Affiliations:** 1 Department of Gastroenterology and Hepatology, Gazi University, Ankara 06560, Turkey; dr.k.moral@gmail.com (K.M.); drmehmetcindoruk@gmail.com (M.C.); 2 Department of Gastroenterology and Liver Transplantation, Güven Hospital, Ankara 06540, Turkey; 3 Department of General Surgery, Güven Hospital, Ankara 06540, Turkey; muzafferatli656@hotmail.com (M.A.); sedatkarademir@gmail.com (S.K.); 4 Department of Infectious Disease and Clinical Microbiology, Güven Hospital, Ankara 06540, Turkey; yasarb44@hotmail.com (Y.B.); yesim.sardan@guven.com.trt (Y.S.)

**Keywords:** liver transplantation, immunosupression, prophylaxis, invasive fungal infection

## Abstract

**Background:** Invasive fungal infections (IFIs) after liver transplantation (LT) remain a concern. No universal protocol for antifungal prophylaxis in LT exists. Antifungal prophylaxis varies across European centers. Studies suggest risk stratification for prophylaxis. This study assessed IFI frequency and outcomes in adult LT recipients without antifungal prophylaxis and evaluated risk stratification for predicting IFIs. **Method:** A retrospective analysis of clinical and microbiological data from 244 liver transplant patients focused on IFI within 100 days post-transplantation. Of these, 225 (92%) had right liver transplants from living donors. We assessed two risk stratification models for predicting IFI: one categorizes patients into low- and high-risk groups, and the other divides patients into three categories, with two eligible for prophylaxis and one not. **Results:** Of 244 patients, 3% (seven individuals) developed invasive fungal infections (IFI), including two aspergillosis and five candidiasis. IFI occurred in 8% of high-risk and 2% of low-risk patients in the first stratification, with no significant difference between groups (*p* = 0.144). In the second stratification, IFI was found in 4% of the target and 2% of non-target groups, without a significant difference (*p* = 0.455). Patients with IFI showed higher mean MELD scores of 21.71 ± 2.35 versus 17.04 ± 6.48 in those without IFI (*p* < 0.05). **Conclusions:** This study evaluated IFI outcomes without systemic antifungal prophylaxis in LT recipients. Limited antifungal use in a major living liver donor transplantation (LDLT) group, with low MELD scores and immunosuppression protocols, could be feasible. Future multicenter studies can improve understanding and develop prophylaxis algorithms for LT settings.

## 1. Introduction

The incidence of invasive fungal infections (IFIs), particularly invasive candidiasis and aspergillosis, following liver transplantation (LT) varies from 6% to 47% [1,2]. IFIs are predominantly caused by *Candida* species (60–80%), infections due to Aspergillus (1–8%), and Cryptococcus are less common in clinical practice [3,4,5]. However, the epidemiology of IFI has changed; recent experience suggests that the overall incidence of this complication has declined because of better patient selection, improved surgical techniques, use of lower intensity immunosuppression, and antifungal prophylaxis. The latter is recommended for a subgroup of high-risk LT patients, mostly those with bilio-enteric anastomosis, re-transplanted patients, and those with renal failure [3,6]. While guidelines recommend a targeted prophylaxis approach in clinical practice, specifically, withholding prophylaxis for low-risk patients and administering it to high-risk patients, there is no universal standard for antifungal prophylaxis practices among transplant centers [5,7]. According to a recent survey from Europe, antifungal prophylaxis was administered to all LT recipients (universal prophylaxis) in 35% of centers, to patients at high risk in 53% of centers, and no prophylaxis was given in 12% of centers [1]. Various models for risk stratification have been developed for the selection of high-risk patients and orienting the targeted antifungal prophylaxis in the clinical setting [8,9]. Here, we aimed to analyze the incidence and outcome of invasive fungal infections in adult LT recipients who received no systemic antifungal prophylaxis and assessed the feasibility and efficacy of risk stratifications in predicting IFIs in a single center.

## 2. Materials and Methods

### 2.1. Study Population

Clinical and microbiological data of consecutive 248 LT patients between 1 February 2014–31 December 2023 not exposed to systemic antifungal prophylaxis were retrospectively reviewed. Six patients who died within five days after transplantation were excluded from the study. Given that the majority of IFIs occur within the first 3 months due to heightened immunosuppression, we monitored IFIs for a period of 100 days [10]. To monitor IFI within 100 days post-transplantation, two patients who underwent re-transplantation at 150 days and 1071 days following their initial transplantation due to recurrence of the primary disease were considered and analyzed as distinct cases for the second transplantation, resulting in a total of 244 liver transplants (LTs). Ethical approval was given by the Gazi University Ethical Commission Board.

### 2.2. Immunosuppressive Protocol

The immunosuppressive protocol consisted of a low-dose tacrolimus (tac) based triple regimen. Tac was administered at a starting dose of 0.05 mg/kg/day in two divided doses to target whole-blood trough levels of 8–10 ng/mL for the first three months after transplantation, 6–8 ng/mL in the rest of first year, and 3–5 ng/mL, thereafter. Mycophenolate mofetil administration was started within 24 h of transplantation at doses of 1.5 g/day (in two divided doses) and discontinued 6–12 months post-LT. The corticosteroid regimen consisted of methylprednisolone after the intraoperative induction, at a dose of 100 mg/day on day 1, tapered to a maintenance dose of 15 mg/day by day 10, and discontinued by the 3rd month post, except for the cases with autoimmune etiology. Anti-thymocyte globulin at doses of 1.5–3 mg/kg/day was given as induction therapy in two combined liver and kidney transplants from cadaveric donors. Acute cellular rejection episodes were treated with bolus intravenous corticosteroid therapy using 2 g of methylprednisolone.

### 2.3. Infectious Disease Management

As a member of a multidisciplinary team, the infectious disease specialist is involved in the management of all liver transplant recipients with suspected or confirmed infections. Piperacillin combined with Tazobactam was administered as surgical prophylaxis for a maximum duration of 24 h. No systemic antifungal or anti-Pneumocystis pneumonia prophylaxis was provided. Recipients were scheduled to receive nystatin at a dosage of 50,000 units three times daily as prophylaxis against mucosal candidiasis for the first three months. Oral nystatin is not considered a systemic antifungal due to its lack of absorption from the gastrointestinal tract [11].

Empirical broad-spectrum antimicrobial treatment was initiated for the management of infectious episodes following the acquisition of appropriate cultures. Treatment was subsequently adjusted based on the identification of the specific etiological agent and its antimicrobial susceptibility profile. Intravenous fluconazole was the antifungal agent of choice for empirical therapy. Depending on the results, echinocandins or liposomal amphotericin B were employed if necessary. Three months of anti-cytomegalovirus prophylaxis with valganciclovir was administered, commencing after the first week post-liver transplantation.

In line with the purpose of this study, fungal infection in our patients was retrospectively defined according to the revised EORTC/MSGERC definition for Invasive Fungal Infections [12].

### 2.4. Definition of Risk Stratification

**Figure 1:**
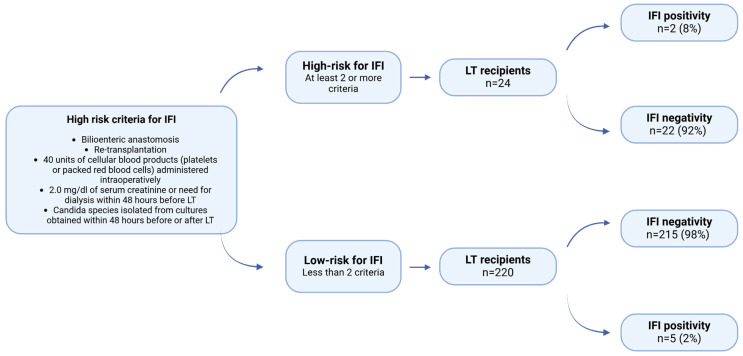
Distribution of patients to high-risk and low-risk for IFI according to the proposed risk stratification by Pappas et al. [8] (LT = Liver transplantation).

Two risk stratifications were used to assess their utility and usefulness in the clinical setting. The first risk stratification classifies patients into low- and high-risk for IFI [8]. Patients who fulfill at least two high-risk criteria are categorized as high-risk for IFI and should receive antifungal prophylaxis. Conversely, if patients meet fewer than two high-risk criteria, antifungal prophylaxis is not recommended (Figure 1).

**Figure 2:**
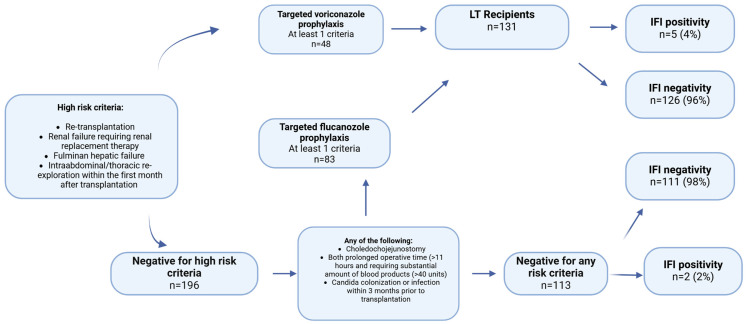
Distribution of patients to target and non-target groups according to the proposed risk stratification by Eschenauer et al. [9] (LT = Liver transplantation).

The second risk stratification, as adapted from the American Society of Transplantation (AST) and the Infectious Diseases Society of America (IDSA), categorizes patients into three groups: two target groups eligible for prophylaxis and one non-target group ineligible for prophylaxis. Patients presenting with at least one high-risk criterion, such as retransplantation, renal failure necessitating replacement therapy, fulminant hepatic failure, or intra-abdominal/thoracic re-exploration within the first month post-transplantation, should be administered voriconazole prophylaxis. In the absence of high-risk criteria, if at least one criterion from the “any of the following” category is present, fluconazole should be administered. If neither condition is met, the patient should not receive any prophylaxis. The voriconazole and fluconazole target groups were combined to form the total target group (Figure 2) [9].

We applied both stratifications to assess their significance and efficacy in routine clinical practice within our liver transplant (LT) cohort.

### 2.5. Statistical Analysis

Data analysis was performed using IBM SPSS Statistics for Windows, version 26.0, (IBM Corp., Armonk, NY, USA). Categorical variables were defined as numbers and percentages, whereas normally distributed variables were expressed as means (±standard deviation). For non—parametrically distributed variables, the median (min-max) and range of 25–75% were used. Chi-square was employed for the analysis of the relationship between categorical variables. An independent samples t-test was used to compare two sample means from unrelated groups. Factors that had a *p* < 0.20 in the univariate analysis were put into the multivariate analysis to assess the correlation between IFI development and clinical factors.

## 3. Results

The demographic and perioperative data of the patients are presented in Table 1.

All patients were followed for at least 112 days. In 225 (92%) of 244 LT patients, right liver grafts were transplanted from living donors. Piggy-back was used as a routine technique in all cases, including cadaveric transplants.

### 3.1. Incidence of Fungal Infection and Empirical Antifungal Therapy

Within 100 days of surgery, 42 patients (17%) received empirical antifungal treatment along with antibiotics. In 36 of these, fluconazole was administered, while caspofungin was administered in the remaining six. The average initiation day of antifungal therapy was eight days, ranging from 1 to 41 days. Tests for fungal infection were reported as positive in 14 (33%) patients, while the remaining 28 (67%) yielded negative results in the patients who were initiated on empirical antifungal therapy. Among the 14 patients with positive cultures, seven (50%) were diagnosed with IFI. *Aspergillus* species were identified in two cases, one isolated from cerebrospinal fluid (CSF) and another from deep tracheal aspirate (DTA) (Table 2).

*Candida albicans* was detected in four patients: three samples from the abdominal collection who were diagnosed with abdominal sepsis, and one blood culture from patients with candiduria. Additionally, *Candida glabrata* was yielded in a wound culture from one patient. Except for one patient with pulmonary aspergillosis who died, all the remaining IFIs recovered after treatment and survived the infection. Of these six survivors, two died 155 and 807 days after transplantation due to non-infectious causes, respectively.

### 3.2. Analysis of the Risk Stratifications

Of the 244 patients, seven were found to have IFI (3%). Among 24 patients in the high-risk group, 2 (8%) patients developed IFI, while 22 (92%) patients did not. In contrast, 5 (2%) patients in the low-risk group had IFI, and 215 (98%) patients didn’t have IFI. There was no statistically significant relationship between risk stratification and IFI occurrence (*p* = 0.144) (Figure 1).

Among the patients in the target group, 5 (4%) developed IFI, while 126 (96%) did not. In contrast, among those in the non-target group, 2 patients (2%) had IFI, and 111 patients (98%) did not. There was no statistically significant relationship between the target group and the presence of IFI (*p* = 0.455) (Figure 2).

### 3.3. Difference in MELD Scores and Intensive Care Unit (ICU) Between IFI and Non-IFI Groups

The biological model for end-stage liver disease (MELD) score varied substantially based on IFI status (*p* < 0.05). Patients with IFI had a mean MELD of 21.71 ± 2.35, whereas those without IFI had a mean MELD of 17.04 ± 6.48. Consequently, the average biological MELD for patients with IFI was significantly higher. The number of intensive care unit (ICU) days was statistically significantly different between the invasive fungal infection (IFI) and non-IFI groups (*p* < 0.05). The proportion of patients who remained in the ICU for more than 48 days was 86% in the IFI group, compared to 25% in the non-IFI group. No significant differences were observed in the subgroup analysis concerning age, non-tumoral/tumoral etiology, bilioenteric anastomosis, cold-ischemia time, operation time, volume of blood transfusion, renal replacement therapy, and biliary leaks (Table 3).

### 3.4. Univariate and Multivariate Analysis

While there was a difference regarding the IFI and non-IFI groups in terms of MELD score and intensive care unit days *p* < 0.005 in the subgroup analysis, only in the univariate analysis did the intensive care unit days positively correlate with the development of IFI (*p* < 0.05). However, in the multivariate analysis, no statistically significant correlation was found (Table 4).

### 3.5. Mortality

Twenty-two transplant recipients died within 100 days after their procedure (with a median of 17 days and a range of 6 to 98 days). Of these 22 patients, seven were administered empirical antifungal therapy. Of these seven patients, only one died due to invasive aspergillosis, while the remaining six were not diagnosed as either invasive or noninvasive fungal infections. Among the patients, five were identified as belonging to the target group and were thus eligible for antifungal prophylaxis. In contrast, only three patients were classified as high-risk according to the first risk stratification. Notably, the patient who developed invasive fungal infection (IFI) was categorized as belonging to the low/non-target group. In summary, only one of the 22 patients who died within 100 days after transplantation developed an invasive fungal infection.

## 4. Discussion

Effective management of IFI in the early post-transplant period is essential because IFI has a high overall mortality rate in these patients [13,14]. According to AST/IDSA, antifungal prophylaxis is a crucial aspect of managing IFI. The AST/IDSA suggests that all adult liver transplant recipients who are at a high risk of developing IFI should receive targeted prophylaxis. The risk factors identified by the AST/IDSA include re-transplantation, re-operation, renal failure requiring hemodialysis, transfusion of ≥40 units of cellular blood products including platelets, packed red blood cells, and auto-transfusion, choledocho-jejunostomy, and *Candida* colonization in the perioperative period [15]. Other risk factors identified that warrant prophylaxis in different studies were biliary leaks and living donor transplants [9,16].

The most common fungal pathogens in liver transplant recipients are *Candida* species, which are responsible for more than 80% of the invasive fungal infections (IFIs) in this group [17,18]. Infections caused by *Aspergillus* species, other molds, and *Cryptococcus neoformans* are less common but are still significant pathogens in the post-transplant period. The vast majority of IFIs occur within two months after orthotopic liver transplantation (OLT) [19,20]. The results of our center regarding the cause and onset of fungal infection are in accordance with the literature; 12 of the 14 patients had fungal infection with *Candida* species, while two patients were infected with *Aspergillus* species. Among these fungal infections, seven were identified as IFI (Table 2).

The initial risk stratification proposed by P.G. Pappas et al. [8] recommended antifungal prophylaxis for high-risk individuals. Upon implementation of this risk stratification in our patient population, it was observed that 2 (8%) of the high-risk patients (n = 24) developed IFI, while the remaining 22 (92%) did not. The first risk stratification was more precise in not giving any antifungal prophylaxis to a total of 220 patients, but 22 patients would be given unnecessary prophylaxis, and five patients would develop IFI in the low-risk group (Figure 1). No statistically significant association was found between risk stratification and IFI occurrence (*p* = 0.144).

The second risk stratification was less precise in predicting the development of IFI. The prevalence of IFI was 4%, whereas 96% did not exhibit IFI in the target group (n = 131). In the non-target group (n = 113), 2 patients developed only IFI. A total of 131 patients would be given unnecessary prophylaxis. The second risk stratification did not reveal a statistically significant relationship between the target group and presence of IFI (*p* = 0.144) (Table 4). The study also found that living liver donor transplantation (LDLT) and biliary leaks are risk factors for IFI. In our research, only three out of the forty patients with biliary leaks had IFI, and only seven out of 225 living liver transplant recipients had IFI (Table 1). The increased risk of fungal infections attributed to LDLT in the previous study may be due to a lack of experience in the field of LDLT, leading to longer operation times and increased complications. Most of our transplants involve LDLT, so increased experience and fewer complications may be the cause for the lower incidence of IFIs in comparison to Western centers, where LDLT is less commonly performed. Two studies, one involving 554 and another analyzing 190 LT patients, have identified bilioenteric anastomosis as a risk factor for the development of IFI [21,22]. However, in our study, neither the subgroup analysis nor the multivariate analyses identified bilioenteric anastomosis as a risk factor for IFI development. This discrepancy may be attributed to differences in the settings of living donor liver transplantation (LDLT) and deceased donor liver transplantation (DDLT), as well as the elective nature of our surgical procedures. The primary influential factor may be the MELD score. At our center, the median MELD score was 17.8, and the majority of our patients underwent elective surgery, which may have contributed to the reduced risk of fungal infection. A retrospective single-center study conducted by Faouzi Saliba involving 667 liver transplant recipients examined the incidence of fungal infections and identified potential risk factors. The multivariate analysis revealed that a MELD score of 20–30 or ≥30 was associated with a 2.0-fold or 4.3-fold increased relative risk of fungal infection and a 2.1-fold or 3.1-fold increased relative risk of invasive fungal infection, respectively. The study concluded that patients with a MELD score ≥ 20, particularly those with a score ≥ 30, should be considered for antifungal prophylaxis [16]. Another study conducted by Utsumu et al., who analyzed 153 living liver donor recipients, found that a MELD score of ≥26 was associated with an increased risk of IFI [23]. In our research, patients with IFI displayed a mean MELD score of 21.71 ± 2.35, while those without IFI had a mean score of 17.04 ± 6.48. The average biological MELD score for individuals with IFI was significantly higher (*p* < 0.005) than that of those without IFI. However, in the logistic regression analysis, only the number of ICU days was positively correlated with the development of IFI in the univariate analysis, whereas the MELD score was not. This could be due to the limited number of patients who developed IFI, which may have affected the reliability of the logistic regression analysis compared to the comparison of MELD scores between IFI and non-IFI patients. Even though there is no cut-off value for MELD for IFI development, it can be concluded that the sicker patients with relatively higher MELD scores have higher IFI risk. Neither the initial nor the secondary risk stratification incorporated MELD scores in their risk evaluation. Although the initial stratification demonstrates greater efficacy in predicting IFI outcomes than secondary stratification, employing targeted antifungal prophylaxis based on MELD scores could potentially be more practical. This approach may be particularly advantageous in the LDLT context, where transplantations are typically conducted electively and often in outpatient settings. The use of MELD scores for targeted antifungal prophylaxis could potentially result in more efficient resource allocation and improved patient outcomes in LDLT settings.

Environmental exposure, as well as the characteristics of the patient’s immune system and colonization, pose a risk for invasive fungal infections. Infection control measures were meticulously implemented during renovation or construction work at our hospital. Additionally, antibiotic use and duration were determined by specialists in infectious diseases. Antimicrobial stewardship principles were followed according to the protocol. This may have contributed to the low risk of fungal colonization or infection.

Although we administered empirical antifungal treatment in conjunction with antibiotics to 42 patients, this therapy was discontinued immediately in the clinical setting once cultures turned negative or when clinical suspicion was removed. By choosing not to administer long-term prophylaxis, we lowered the chances of liver-related toxicity and reduced drug-drug interactions, particularly those linked to azole antifungals, while also decreasing the likelihood of developing resistance to antifungal agents [4,24]. Recommendations for the use of antifungal prophylaxis in solid organ transplantation are largely based on single-center studies, anecdotal experiences, and reviews from transplant centers. Additionally, data from other patients, such as those with hematological malignancies and hematopoietic stem cell transplants (HSCTs), provide further insight into these infections [25,26,27]. The immunosuppressive protocol after liver transplantation is not as intense as renal transplantation or HSCT. This offers the advantage of easier management and prophylaxis for infectious complications. Extrapolation of practices creates conflicting results regarding antifungal usage and fungal infections in liver recipients, as reported in the literature [28,29]. For this reason, there should be larger prospective studies determining antifungal usage and fungal infection risk stratification, including both DDLT and LDLT from centers across the globe. The limitations of our study are the single-center experience, the limited number of patients having IFI, and the lack of a control group of patients undergoing DDLT. Future research should focus on developing standardized protocols for antifungal prophylaxis in liver transplant recipients, considering both DDLT and LDLT procedures. Multi-center collaborations could provide valuable insights into regional variations in fungal infection rates and risk factors. Additionally, long-term follow-up studies are required to assess the impact of different antifungal strategies on patient outcomes and graft survival.

## 5. Conclusions

In conclusion, this study highlights the need for further research to address the limitations encountered and to enhance our understanding of antifungal prophylaxis in liver transplantation. By developing standardized protocols and conducting multi-center collaborations, the transplant centers can improve patient care and outcomes for LT recipients. Long-term follow-up studies are needed to evaluate the efficacy of various antifungal strategies in LT.

## Figures and Tables

**Table 1:** Patient characteristics.

Characteristic	Cohort of Patients (N = 244)
Age, mean (std)	57 ± 11 *
Male, n (%)	172 (71%)
MELD ^†^, mean (std)	17.8 (6–40) **
Etiology, n (%)	
Non-Tumor	166 (68%)
With Tumor HCC	78 (32%) 74 (30%)
Perioperative variables	
CIT, min	80 (40–632) **
Op time, min	502 (185–950) **
PRBC, units	3.2 ± 3.1
ICU stay, days	1 (1–30) **
ICU stay > 48 h, n (%)	66 (27%)
Post-Tx RRT (within 1st mon), n (%)	9 (4%)
Biliary leak n (%)	40 (16%)
Amount of IFI in BL group	3 (7%)
Type of anastomosis	
Duct to duct n (%)	132 (54%)
Hepaticojejunostomoy n (%)	112 (46%)
LDLT n (%)	225 (92%)

* (Mean ± standard deviation) ** (Median 25–75%). MELD = model of end-stage liver disease, † biologic MELD, CIT = cold ischemia time, PRBC = packed red blood cells, RRT = renal replacement therapy, IFI = invasive fungal infection, BL = Biliary leak.

**Table 2:** Clinical and microbiological data from 14 LT recipients with positive culture or histopathologic features of fungi.

	LT No	Antifungal Treatment (Empirical)	POD	Organism	Site	Risk of IFI L/H * 1 * Risk Stratification	Risk of IFI T/NT ** 2 * Risk Stratification	IFI	Survival
1	15	Fluconazole	97	*C. albicans*	Esophagus	L	NT	-	Alive
2	17	Fluconazole	33	*C. glabrata*	Wound (abdominal sepsis)	High	T	Yes	Alive
3	47	Fluconazole	12	*C. albicans*	Urine (+Foley catheter)	L	T	-	Alive
4	65	Fluconazole	18	*C. albicans*	Peritoneal fluid (JP drain)	L	T	-	Alive
5	82	Fluconazole	7	*C. albicans*	Urine (+Foley catheter)	L	NT	Yes	Alive
6	83	Flucanozole > Voriconazole	62	*Aspergillus* spp.	CSF	L	T	Yes	Alive
7	117	Fluconazole	7	*C. albicans*	Peritoneal fluid (JP drain)	L	T	-	Alive
8	128	Fluconazole	4	*C. albicans*	Wound (accepted as colonization)	High	T	-	Died (reLT)
9	172	Fluconazole > Voriconozale > Amphotericin B	4	*Aspergillus* spp.	Endotracheal aspirate culture	Low	T	Yes	Died
10	176	Caspofungin > Fluconazole	9	*C. albicans*	Peritoneal fluid (JP drain)	High	T	Yes	Died
11	186	Caspofungin	8	*C. albicans*	Peritoneal fluid (JP drain)	L	T	-	Alive
12	194	Flucanozole > Caspofungin	9	*C. albicans*	Peritoneal fluid (JP drain)	L	T	Yes	Died
13	221	Caspofungin	7	*C. albicans*	Peritoneal fluid (JP drain)	L	NT	Yes	Alive
14	245	Flucanozole > Caspofungin	5	*C. albicans*	Wound	L	T	-	Alive

IFI = invasive fungal infection, * L/H = Low/High, ** T/NT = Target/Non-Target Group, POD = postoperative day, JP = Jackson-Pratt, CSF: cerebrospinal fluid.

**Table 3:** Comparison of baseline characteristics of IFI and Non-IFI groups.

	IFI Group (n = 7)	Non-IFI Group (n = 237)	*p*-Value
Age mean (std)	56 ± 11 *	57 ± 10 *	*p* = 0.513
Male n (%)	5 (71%)	167 (71%)	*p* = 0.413
MELD	21.71 ± 2.35 *	17.04 ± 6.48 *	*p* = 0.004
Etiology n (%) Non-Tumor With Tumor HCC	3 (43%) 4 (57%) 4 (57%)	163 (69%) 74 (31%) 70 (30%)	*p* = 0.314 *p* = 0.300 *p* = 0.295
Perioperative variables CIT, min Op time, min PRBC, units ICU stay, days ICU stay > 48 h n (%)	71 (50–610) ** 386 (386–950) ** 3.0 ± 2.9 * 4 (2–8) ** 6 (86%)	80 (40–632) ** 502 (185–950) ** 3.1 ± 2.9 * 1 (1–3) ** 59 (25%)	*p* = 0.310 *p* = 0.080 *p* = 0.252 *p* = 0.004 *p* < 0.001
Post-Tx RRT (within 1st mon), n (%)	0 (0%)	7 (0.03%)	*p* = 0.645
Biliary leak n (%)	3 (43%)	37 (16%)	*p* = 0.07
Type of anastomosis Duct to duct n (%) Hepaticojejunostomoy n (%)	3 (43%) 4 (57%)	129 (54%) 108 (46%)	*p* = 0.530 *p* = 0.510

* (Mean ± standard deviation) ** (Median 25–75%). MELD = model of end-stage liver disease, CIT = cold ischemia time, PRBC = packed red blood cells, RRT = renal replacement therapy, IFI = invasive fungal infection, BL = Biliary leak.

**Table 4:** Univariate and Multivariate Analysis for IFI development.

	Univariate Analysis 95 %CI				Multivariate Analysis 95 %CI			
	OR	Lower	Upper	*p*	OR	Lower	Upper	*p*
ICU days	1.141	1.033	1.262	0.010	1.082	0.951	1.230	0.232
DM	1.967	0.197	19.5	0.564	-	-	-	-
Age	1.050	0.956	1.152	0.306	-	-		-
MELD	1.114	0.990	1.254	0.073	1.055	0.944	1.179	0.346
Retransplantation	0	0	-	0.999	-	-	-	-
Reoperation	1.20	0.887	1.532	0.460	-	-	-	-
Blood transfusion >40	0	0	-	0.999	-	-	-	-
Pretransplant Cre > 2 or need for hemodialysis pretransplant	2.82	0.200	39.2	0.443	-	-	-	-
Fulminan hepatic failure	0	0	-	0.999	-	-	-	-
Bilioenteric anastomosis	1.31	0.262	6.64	0.737	-	-	-	-
Biliary Leaks	0.846	0.099	7.225	0.879	-	-	-	-

IFI = Invasive fungal infection, ICU (Intensive care unit), DM (Diabetes Mellitus), Cre (Creatinine).

## Data Availability

The datasets analyzed during the current study are available from the corresponding author on reasonable request.

## Abbreviations

IFI	Invasive fungal infection
LT	Liver Transplantation
LTs	Liver transplants
tac	Tacrolimus
AST	American Society of Transplantation
IDSA	Infectious Diseases Society of America
CSF	Cerebrospinal fluid
DTA	Deep tracheal aspirate
MELD	Model For End Stage Liver Disease
LDLT	Living Liver Donor Transplantation
HSCTs	Hematopoietic stem cell transplants
DDLT	Deceased Donor Liver Transplantation
